# Frontiers and advances in environmental soil chemistry: a special issue in honor of Prof. Donald L. Sparks

**DOI:** 10.1186/s12932-020-00070-y

**Published:** 2020-04-17

**Authors:** Young-Shin Jun, Mengqiang Zhu, Derek Peak

**Affiliations:** 1 Department of Energy, Environmental & Chemical Engineering, Washington University, St. Louis, MO 63130 USA; 2 Department of Ecosystem Science and Management, University of Wyoming, Laramie, WY 82071 USA; 3 Department of Soil Science, University of Saskatchewan, Saskatoon, SK S7N 5A8 Canada

**Figure:**
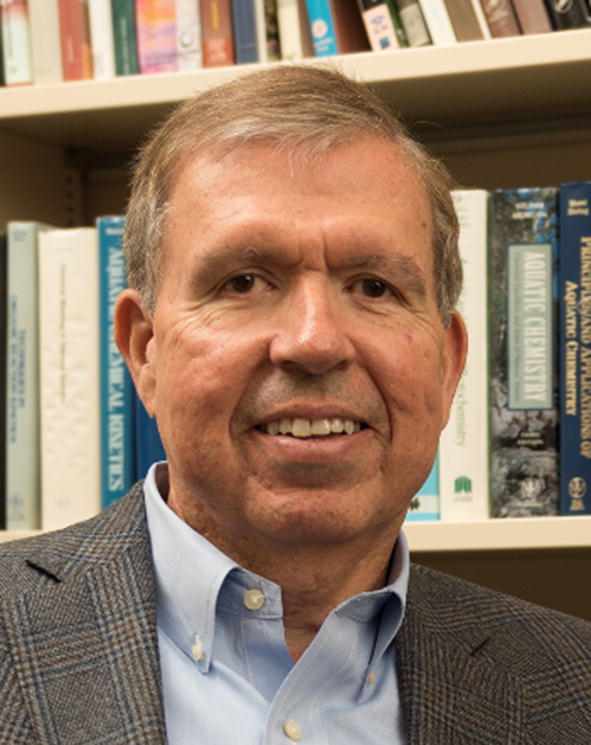
Professor Donald Sparks

This Special Issue of *Geochemical Transactions* is dedicated to Professor Donald L. Sparks, the S. Hallock du Pont Chair in the Department of Plant and Soil Sciences, and the Director of the Delaware Environmental Institute, at the University of Delaware (UD), in celebration and honor of his life-long research interests and achievements in environmental soil chemistry. Dr. Sparks is the recipient of the 2015 American Chemical Society’s Geochemistry Division Medal for his highly influential and transformative work in soil and environmental geochemistry, his outstanding record as an educator and mentor, and his service to the geochemical community. Dr. Sparks received his B.S. in Agronomy in 1975 and M.S. in Soil Science in 1976, both from the University of Kentucky, before he obtained his Ph.D. in Soil Science in 1979 from Virginia Polytechnic Institute and State University. Dr. Sparks has received numerous awards, including UD’s Francis Alison Award, the Liebig Medal from the International Union of Soil Sciences, the U.S. Department of Agriculture’s Sterling Hendricks medal, the Northeast Association of Graduate Schools Geoffrey Marshall Mentoring Award, the Soil Science Research Award, the M.L. and Chrystie M. Jackson Soil Science Award, and the American Society of Agronomy’s Environmental Quality Award.

Over the past 30 years, Dr. Sparks’ research has fundamentally transformed our understanding of the fate of toxic metals and plant nutrients in soils, and of chemical reactions at solid-water interfaces. His research utilizes synchrotron X-ray and other spectroscopic techniques to determine the forms of the metals and nutrients in the soil at the molecular scale, revealing how they interact with mineral surfaces and accumulate in plants. Such information determines the mobility, toxicity, and bioavailability of contaminants in the soil and is useful in developing effective strategies for soil remediation. As we began to plan this honorary Special Issue, we invited experts and colleagues who share this scientific sphere with Dr. Sparks. The resulting Special Issue highlights important challenges in environmental geochemistry and soil chemistry and introduces current advances in these areas. We have also brought together a series of research articles exemplifying recent developments in state-of-the-art experimental and computational approaches to understanding mineral–water interfaces.

The Special Issue starts with Dr. Sparks’ feature article, which introduces the increasing importance of soil chemistry in climate change and in critical soil interactions with nutrients and emerging organic contaminants such as antibiotics, hormones, and per- and polyfluoroalkyl substances (PFAS). Dr. Sparks has provided future research directions as well as challenges and opportunity in environmental soil chemistry [1]. In soils, two important reactive elements are iron and manganese (hydr)oxides. The contributed articles provide interesting examples of these highly reactive minerals in soils and highlight the importance of understanding them at the molecular scale: Voegelin et al. examine the reductive dissolution kinetics of an environmentally relevant set of arsenate-containing Fe(III)-precipitates whose structure changes as a function of phosphate (P) and silicate (Si) content in its structure [2]. Schaefer et al., characterize the reaction of aqueous Fe(II) with pyrolusite (β-MnO_2_), using electron microscopy, X-ray diffraction, aqueous Fe and Mn analyses, and ^57^Fe Mössbauer spectroscopy, and describe the continuous redox chemistry between Fe(II) and Mn/Fe oxides [3]. By experimental analysis and density functional theory (DFT) calculations, Kubicki et al. simulate interactions between chromate and a ferrihydrate nanoparticle [4]. Then, several articles by Sowers et al. [5], Stuckey et al. [6], Sundman et al. [7], and Zhu et al. [8] discuss how iron and manganese (hydr)oxides interact with organic compounds undergoing redox reactions and dissolution. In addition, Cade-Menun et al. [9] and Hamilton et al. [10] investigate the fate and transport of phosphate as a nutrient in soil systems by using inductively coupled plasma spectroscopy, P-nuclear magnetic resonance, and X-ray absorption spectroscopy. Strawn et al. provide a nice review that discusses phosphorus and arsenic in soil using four case studies [11]. Nickel, zinc, and copper in soils are trace transition metals and critical nutrients as well. Using microfocused X-ray fluorescence, diffraction, and absorption spectroscopy, Siebecker et al. report natural speciation of nickel in serpentine topsoils [12] and Gou et al. report a competitive adsorption of nickel and zinc on aluminum oxides [13]. Fan et al. measure the Wien effect in colloidal suspensions containing cadmium and zinc to determine binding energies associated with cadmium and zinc ion adsorption in clay-containing soils [14]. In addition to macroscale colloids, soil contains many nanosized pore spaces. Knight et al. discuss nanoscale confinement effects on copper ion adsorption on mesoporous silica and highlight the important unique nanoscale nature of pores in soil particulates [15]. Furthermore, engineered nanomaterials can also enter natural soil environments and become incidental soil components, but their impacts on the environment are poorly known. To pursue this aspect, Zeng et al., study CuO nanoparticles and their catalytic behavior in the presence of arsenic, using in situ quick scanning X-ray absorption spectroscopy (Q-XAS) analysis [16]. Another example incidental nanoparticle is spinel, Zn-bearing magnetite (Zn_0.5_Fe_2.5_O_4_) and minium (Pb_3_O_4_), that were found in proximity to a former Cu-smelter in Timmins, Ontario, Canada [17]. The Special Issue covers a wide variety of transition metals, organic matter, nutrients, toxins, and soil components and introduces studies enabled by the most advanced X-ray techniques, NMR, and high-resolution transmission electron microscopy. The exciting discussions provide macro to nanometer-scale insights into soil systems and exemplify the topics that Dr. Sparks has pursued throughout his career.

Thinking back to our first solicitations for the Special Issue, we were impressed by the enthusiasm from the geochemical society, which reflects Dr. Sparks’ dedication and leadership in environmental soil chemistry. We are grateful to have this support from our colleagues and excited to share this Special Issue. To facilitate its dissemination, Mr. Samuel Winthrop and Mr. Jan Margulies, Journal Development Editors of *Geochemical Transactions*, and Dr. Sherestha Saini, Senior Editor of Springer Nature’s Environmental Sciences Journals, have kindly helped in handling the manuscripts. We hope that this Special Issue will reach the broader environmental soil geochemistry community. Lastly, thank you, Dr. Sparks, for your leadership in environmental soil chemistry and for inspiring many of us.
